# Kindlin-2 Modulates the Survival, Differentiation, and Migration of Induced Pluripotent Cell-Derived Mesenchymal Stromal Cells

**DOI:** 10.1155/2017/7316354

**Published:** 2017-01-09

**Authors:** Mohsen Moslem, Reto Eggenschwiler, Christian Wichmann, Raymund Buhmann, Tobias Cantz, Reinhard Henschler

**Affiliations:** ^1^Department of Transfusion Medicine, Cell Therapeutics and Hemostaseology, Ludwig-Maximilians University Hospital, Munich, Germany; ^2^Translational Hepatology and Stem Cell Biology, REBIRTH Cluster of Excellence and Department of Gastroenterology, Hepatology, and Endocrinology, Hannover Medical School, Hannover, Germany; ^3^Cell and Developmental Biology, Max Planck Institute for Molecular Biomedicine, Münster, Germany; ^4^Blood Transfusion Service, SRC, Zürich, Switzerland; ^5^Blood Transfusion Service, SRC, Chur, Switzerland

## Abstract

Kindlin-2 is a multidomain intracellular protein that can be recruited to *β*-integrin domains to activate signaling, initiate transcriptional programs, and bind to E-cadherin. To explore its involvement in cell fate decisions in mesenchymal cells, we studied the effects of Kindlin-2 modification (overexpression/knockdown) in induced pluripotent cell-derived mesenchymal stromal cells (iPSC-MSCs). Kindlin-2 overexpression resulted in increased proliferation and reduced apoptosis of iPSC-MSCs, as well as inhibition of their differentiation towards osteocytes, adipocytes, and chondrocytes. In contrast, siRNA-mediated Kindlin-2 knockdown induced increased apoptosis and increased differentiation response in iPSC-MSCs. The ability of iPSC-MSCs to adhere to VCAM-1/SDF-1*α* under shear stress and to migrate in a wound scratch assay was significantly increased after Kindlin-2 overexpression. In contrast, inhibition of mixed lymphocyte reaction (MLR) was generally independent of Kindlin-2 modulation in iPSC-MSCs, except for decreased production of interleukin-2 (IL-2) after Kindlin-2 overexpression in iPS-MSCs. Thus, Kindlin-2 upregulates survival, proliferation, stemness, and migration potential in iPSC-MSCs and may therefore be beneficial in optimizing performance of iPSC-MSC in therapies.

## 1. Introduction

 Kindlins are intracellular multidomain proteins with binding motifs that mediate their interaction with integrins, the cytoskeleton, or, in the case of Kindlin-2, E-cadherin [1]. Kindlins can activate integrins by adhering to their *β* cytoplasmic chain using the FERM domain to engage a-actinin, migfilin, or integrin-linked kinase (ILK), which leads to actin remodeling, cell migration, and lamellipodia formation [2]. Kindlin-2 was found to play a role during embryogenesis by altering the proliferation potential and migration behaviour of different cell types, and the deregulation of Kindlin-2 can halt embryonic development and induce embryonic lethality [3]. Kindlin-2 was found to trigger epithelial mesenchymal transition (EMT) by activating Wnt signaling in vitro [4], resulting in increased adhesion, migration, and proliferation [5]. Kindlin-2 may also inhibit the growth and migration of colorectal cancer cells [6]. Because EMT occurs during induced pluripotent stem cells (iPSCs) differentiation towards mesenchymal-like cells [7], we aimed to investigate the role of Kindlin-2 in the functions of iPSC-derived MSC. We hypothesized that Kindlin-2 may increase proliferation, enhance migration and adhesion, and increase functional activation of iPSC-MSCs and thus might provide a basis for engineering iPSC-MSCs in a therapeutically desirable manner.

Obtaining sufficient amounts of MSCs has been a limiting factor for their use in transplantation. Furthermore, the robust functional activation of MSCs, such as migration towards injured tissues, adhesion for homing in areas in need of tissue repair, and resistance to apoptosis after transfusion, was thought to be crucial for therapeutic efficiency in recipients [8, 9]. So far, it is not clear to what extent alterations in the proliferation, migration, and adhesion of therapeutically applied MSCs might influence the capability of the cells to mediate tissue repair or immune regulation. Altogether, “superfunctional” MSCs should display high expandability and survival and boosted adhesion and migration with preserved immunoregulatory properties that are likely to promote the therapeutic potential of MSCs in cellular therapies.

In a previous study, we characterized the differentiation of iPSCs towards MSCs to obtain a functional substitute for ex vivo MSCs [7, 10]. We have shown that iPSCs can be differentiated into MSCs, including development from “epithelial-like” iPSCs towards spindle-shaped MSCs that are capable of proliferation in an undifferentiated stage and of induction into multilineage differentiation. Moreover, iPSC-MSCs showed similar hematopoietic support and immunomodulatory effects to BM-MSCs [10]. In this study, we aimed to modify Kindlin-2 expression in iPSC-MSCs to modulate their proliferative and functional properties. We demonstrate that Kindlin-2 expression levels modulate the adhesion and migration properties of iPSC-MSCs as well as their proliferation, apoptosis, differentiation, and immune-suppression properties.

## 2. Materials and Methods

### 2.1. iPS Cell Culture and Mesenchymal Differentiation

Human iPSCs were provided from in-house supplies as described [11]. Briefly, human fetal liver fibroblasts (FLF) were transduced via lentiviral expression of reprogramming factors Oct4, Sox2, Klf4, and c-Myc (OSKM) and cultured on irradiated mouse embryonic fibroblasts (MEF) in medium containing DMEM/F-12, 20% knockout serum replacement (Thermo Fisher, Waltham, MA, USA), 20 ng/mL human recombinant basic fibroblast growth factor (bFGF, provided from Leibniz University Hannover), 0.1 mM *β*-mercaptoethanol (Thermo Fisher), 1 mM L-glutamine, 1% nonessential amino acids, and 1% penicillin/streptomycin (all from Sigma-Aldrich). Cells were split weekly using collagenase IV (Thermo Fisher), and cells were plated on Matrigel-coated (Corning) plates. Differentiation/enrichment of iPSCs to MSCs was conducted as described [10]. In brief, human iPSC colonies grown on Matrigel were maintained with MSC induction media consisting of DMEM (low-glucose, Sigma-Aldrich, Darmstadt, Germany), 10% defined fetal bovine serum (FBS, Stem Cell Technologies, Vancouver, BC, Canada), 1% nonessential amino acids, 1% penicillin-streptomycin, and 2 ng/mL human recombinant bFGF for 7 days. Next, cells were treated with collagenase IV for 3 min at 37°C, dissociated by glass beads and gentle pipetting, and then passed through 40 mm cell strainers (Fisher Scientific, Schwerte, Germany). Single cells were seeded onto gelatin-coated plates at 1 × 10^4^ cells/cm^2^ in MSC media.

### 2.2. Transfection and Establishment of a Stable Cell Line

The iPSC-MSCs were transfected with four different constructs, including Flag-Kindlin-2 or Flag vector, control short hairpin RNA (shRNA), or Kindlin-2 shRNA. The vectors were received as a gift from Hongquan Zhang, Peking University Health Science Center, Beijing, China. Plasmid structures were described by An et al. [12]. The cells were plated in 6-well plates at a density of 1 × 10^4^ cells/cm^2^ 24 h before transfection. The plasmids were expanded in* Escherichia coli* (*E. coli* strain DH5a) for 16 h and purified by QIAfilter Maxi Kit (Qiagen, Hilden, Germany) following the manufacturer's protocol. The purified plasmid DNA (3 mg/mL) was resuspended in 97.5 *μ*L of low-glucose DMEM, and then 2.5 *μ*L of 0.1 mM polyethylenimine (PEI) was added (Sigma-Aldrich). The PEI/plasmid DNA solution was vortexed immediately to make the PEI/plasmid complexes. The complexes were allowed to interact for 15 min before they were used at room temperature. Then, 600 *μ*L of low-glucose DMEM with 10% FBS was added to the complexes to make a transfection mixture; finally, the mixture was added to the cells. After 3 hours, the medium was removed and replaced with normal MSCs media for 2 days. After two days, cells were cultured under 500 *μ*g/mL G418 (Sigma-Aldrich) selection until all nontransfected cells disappeared.

### 2.3. Proliferation Assays

Cell proliferation assays were performed with WST-1 and BrdU colorimetric assays (both from Roche) according to the manufacturer's protocols. For the WST-1 assay, cells were plated in 96-well plates at an initial density of 2000 cells/well. The growth graphs were made five days after transfection by measuring Formazan dye in the conditioned media. For the quantitative colorimetric BrdU proliferation assay (Roche), BrdU was added 12 hours before fixation. Then, anti-BrdU-POD was added, and the reaction was detected by adding the subsequent substrate. Colorimetric assays were detected with a scanning multiwell spectrophotometer (Bio-Rad). BrdU-incorporated cells were counted five days after transfection. Cells were fixed with 4% paraformaldehyde (PFA) and then subjected to immunofluorescence staining for 5-bromo-2′-deoxyuridine (BrdU) (Abcam). The cells were counted under a fluorescent microscope, and the ratio of BrdU positive nuclei to the total number of nuclei stained with DAPI (Sigma-Aldrich) was determined.

### 2.4. Flow Cytometry

To assess the expression of CXCR-4 after modulation of Kindlin-2 expression, flow cytometric analysis was performed 3 days after transfection as follows: single cell suspensions were prepared by trypsin digestion (Life Technologies) and washed with cold PBS containing 1% bovine serum albumin BSA (Merck Millipore). Next, 2 × 10^5^ cells were incubated for 30 minutes with the respective APC-conjugated monoclonal antibody of CXCR-4 (all from BD Biosciences) and the suspensions were resuspended at a density of 2 × 10^5^ cells per 200 *μ*L in cold PBS containing 1% BSA. Nonspecific fluorescence was determined by incubation of cell aliquots with isotype-matched monoclonal antibody. Samples were run on a FACSCalibur (BD Biosciences, CA, USA) cytometer using FACS Diva software. For each analysis, a minimum of 10,000 cells were assayed. Data were further processed using FlowJo software (Tree Star, Ashland, Oregon, USA).

### 2.5. Flow Chamber Adhesion Assay

For adhesion assays, 10^5^ iPSC-MSCs were allowed to rest for 3 min on a laminar flow chamber slide (*μ*-slide, ibiTreat; IBIDI Systems, Munich, Germany) mounted on an inverted microscope as previously described [13]. Briefly, flow chambers were precoated with 2 *μ*g/mL VCAM-1 fusion protein and cocoated with SDF-1, both from R&D Systems (1 *μ*g/mL). Subsequently, HBSS/0.1% BSA (prewarmed to 37°C) was flushed through the chambers at the indicated calculated shear stresses with increases in steps between 0.35 and 15 dyn/cm^2^ every 30 s. Images were taken, and the adherent cells were counted in four fields for every condition.

### 2.6. Migration Assay

Cells were seeded at a density of 3 × 10^4^ cells on each side of an Ibidi culture insert for live cell analysis (Ibidi, Munich, Germany) with 500 *μ*M separation between each side of the well and were allowed to grow for 24 h. Cells were pretreated with 30 *μ*M mitomycin C for 30 min before removal of the insert, and cells in the insert were incubated in DMEM with or without 30 *μ*M mitomycin C. The cells were photographed using the 10x objective (Zeiss) after insert removal (0 h) and following 24 and 36 h of incubation. Transmigration assays were performed in transwells (Corning, New York, USA) 6.5 mm in diameter with 8 *μ*m pore filters. The upper side of the transwell filter was coated for 1 hour at 37°C with 0.1% bovine gelatin (Sigma-Aldrich) in phosphate-buffered saline (PBS). Then, 5 × 10^5^ transfected iPSC-MSCs suspended in 200 *μ*L of migration medium containing RPMI with 0.25% bovine serum albumin (Sigma-Aldrich) were added to the upper chambers, and 600 *μ*L of migration medium supplemented with 10% FBS was added to the bottom chamber. After 24 h and 48 h incubation of the transwells at 37°C/5% CO_2_, the upper side of the filters was carefully washed with cold PBS, and cells remaining on the upper face of the filters were removed with a cotton wool swab. Transwell filters were stained using a Giemsa solution (Sigma-Aldrich), cut out with a scalpel, and mounted onto glass slides with the lower face turned upwards. The total number of cells that had migrated was counted using light microscopy at 200x magnification. Each experiment was performed in triplicate.

### 2.7. Real-Time PCR

Total cellular RNA was isolated using TRIzol reagent (Life Technologies). Resultant RNA was subjected to DNase treatment and cDNA Synthesis Kit (Life Technologies) with random hexamers. Power SYBR Green Master Mix qRT PCR assays were performed with the StepOne Plus Cycler (Applied Biosystems) using the standard settings. Samples were collected from at least three independent experiments. Kindlin-2 primers used in the real-time PCR were forward sequence 5′-TGTCTCCCCGCTATCTAAAAAAGT-3 and reverse sequence 5′-TGATGGGCCTCCAAGATTCT-3. GAPDH was used as an internal control with the forward sequence 5′-CTGAGAACGGGAAGCTTGT-3 and reverse sequence 5′-GGGTGCTAAGCAGTTGGT-3. Expression of genes was determined using the comparative CT method (2^−ΔΔCT^).

### 2.8. Immune-Suppression Assays

Twenty-four hours after transfection of iPSC-MSCs, mixed lymphocyte reaction (MLR) cultures were inoculated with 5 × 10^4^ mitomycin C-treated (Sigma-Aldrich) human peripheral blood mononuclear cells (PBMCs) as stimulators and 2 × 10^5^ human CD8^+^ T-cells isolated from normal blood donors after informed consent in 96-well round-bottom plates in 200 *μ*L of complete medium containing RPMI 1640 (Life Technologies) supplemented with 0.1 mM *β*-mercaptoethanol, 10% FBS, GlutaMAX I (Life Technologies), 100 U/mL penicillin, and 100 *μ*g/mL streptomycin in the presence or absence of iPSC-MSCs and BM-MSCs as previously described [10]. Cultures were incubated for 5 days in 37°C/5% CO_2_. BrdU was added to the mixed lymphocyte reaction 12 hours before fixation. Then, anti-BrdU-POD was added, and the reaction was detected by adding the subsequent substrate. Colorimetric assays were detected with a scanning multiwell spectrophotometer (Bio-Rad). IL-2 and IFN-*γ* concentrations were determined in MSC/MLR coculture supernatants using commercially available ELISA (BD Biosciences) according to the manufacturer's instructions. Briefly, 50 *μ*L of ELISA diluent was added to the IL-2 or IFN-*γ* coated 96-well plates. Then, 100 *μ*L of sample supernatant or standard controls was added to the wells for 2 hours at room temperature. After washing the samples five times, 100 *μ*L of the prepared working detector was incubated in each well for 1 hour in RT. After washing the samples seven times, 100 *μ*L of TMB substrate was added and incubated for 30 minutes in RT, followed by adding 50 *μ*L of stop solution. Colorimetric assays were detected at 450 nm using a multiwell spectrophotometer (Bio-Rad).

### 2.9. Annexin V Assay

Viability of iPSC-MSCs after transfection was monitored by FACS analysis using annexin V-propidium iodide staining. Cultured iPSC-MSCs were detached, centrifuged, suspended in PBS, and stained with annexin V-FITC and propidium iodide (BD Pharmingen, San Diego, CA, USA). Apoptotic cells were identified as an annexin V-positive/propidium iodide-negative population using the FACSCalibur cytometer (BD) and FlowJo software (Tree Star).

### 2.10. Differentiation of iPSC-MSCs after Kindlin-2 Modification

Differentiation induction of iPSC-MSCs was carried out for 21 days in different differentiation media, 24 hours after transfection. In total, 10^4^ cells were seeded per well in six-well plates (TPP). To induce osteogenic differentiation, cells were cultured with MSC medium containing 1 *μ*M dexamethasone, 0.5 *μ*M ascorbic acid, and 10 mM b-glycerol phosphate (all from Sigma-Aldrich). For adipogenic induction, cells were cultured in MSC medium supplemented with 50 *μ*g/mL indomethacin (Sigma-Aldrich), 50 *μ*g/mL ascorbic acid, and 100 nM dexamethasone. For chondrogenic differentiation, iPSC-MSCs were centrifuged in 0.2 mL of medium at 500 g for 10 min in 15 mL Falcon tubes to form a pellet. The pellets were cultured in MSC medium supplemented with 0.01 *μ*M dexamethasone, 397 *μ*g/mL ascorbic acid-2-phosphate (Sigma-Aldrich), 1 mM sodium pyruvate (Sigma-Aldrich), 10 ng/mL transforming growth factor-*β*1 (TGF-*β*1, Life Technologies), and 1% insulin-transferrin-selenium (Life Technologies). Osteogenesis was assessed by Alizarin Red staining, adipogenesis was assessed by Oil Red-O staining, and chondrogenesis was assessed by Alcian Blue staining (all from Sigma-Aldrich).

### 2.11. Statistical Analysis

The results were expressed as the mean ± standard error of the mean (SEM). Analyses of iPSC-MSCs in vitro were performed using one-way repeated measures analysis of variance (ANOVA) followed by Tukey's post hoc test multiple group comparison to analyze the group differences of the in vivo data. The mean difference was significant at the *p* < 0.05 level. For quantification with ImageJ software, a total of 30 fields of each group were assayed.

## 3. Results

### 3.1. Kindlin-2 Expression Pattern and Targets in iPSC-MSCs

**Figure 1:**
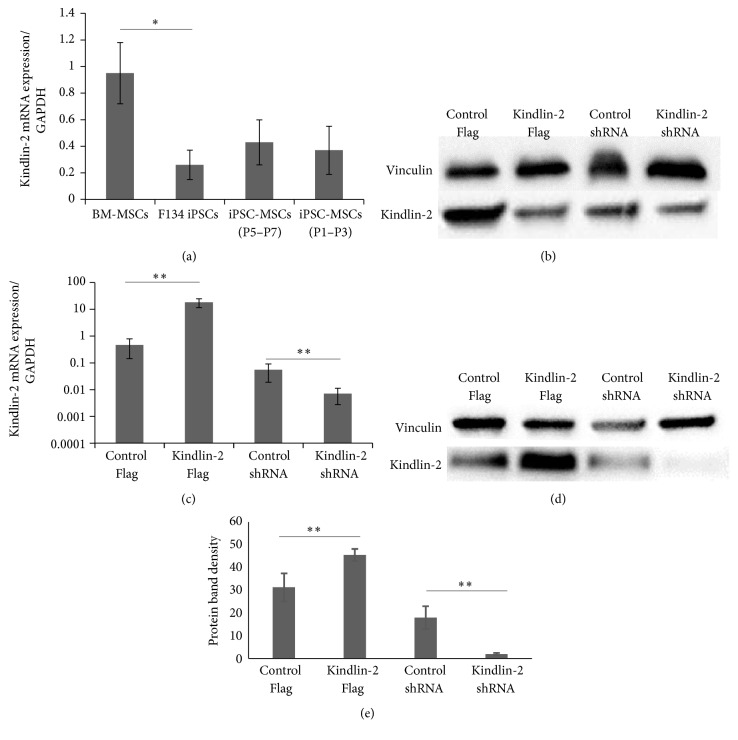
Kindlin-2 expression pattern, overexpression/knockdown, and targets. (a) mRNA expression levels of Kindlin-2 in F134 iPSCs and different passages of iPSC-MSCs indicated significant differences with BM-MSCs; total proteins were extracted from all cell types. (b) Western blotting was performed using an anti-Kindlin-2 monoclonal antibody. (c) Real-time qPCR was performed to quantify mRNA levels of Kindlin-2 in iPSC-MSCs (passages 1–3) with Kindlin-2 overexpression or knockdown. (d, e) Western blot analysis (d) and densitometry (e) performed with ImageJ software were performed and indicate efficient overexpression and knockdown. Data represents the mean expression values normalized to the housekeeping gene GAPDH. ^*∗*^Significance difference *p* ≤ 0.05. ^*∗∗*^Significance difference *p* ≤ 0.01.

As a first approach to assess the role of Kindlin-2 in MSCs, we analyzed its mRNA levels of Kindlin-2 in iPS, BM-MSC, and iPS-MSCs. We found that BM-MSCs express higher levels of Kindlin-2 RNA compared with iPSCs (*p* ≤ 0.05, Figure 1(a)). Different passages of iPSC-MSCs showed a slight increase in mRNA and protein expression levels of Kindlin-2 compared to iPS cells, but still lower than BM-MSCs (Figure 1(b)). For overexpression/knockdown experiments, we used iPSC-MSCs passages 4–6. Quantitative RT-PCR results demonstrated the successful transfection of iPSC-MSCs with Kindlin-2 constructs compared to control plasmids (Figure 1(c)). The corresponding expression of Kindlin-2 protein is shown in Figures 1(d) and 1(e).

### 3.2. Kindlin-2 Promotes Proliferation/Survival and Suppresses Apoptosis of iPSC-MSCs

**Figure 2:**
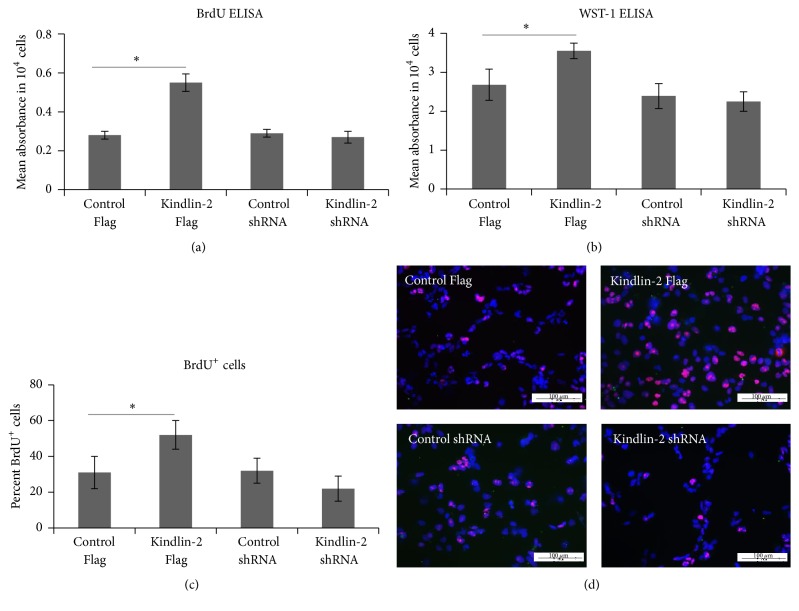
Kindlin-2 regulates iPSC-MSCs cell growth. Cell growth was measured with BrdU (a) and WST-1 (b) ELISA assays and indicated a significantly higher proliferation potential of Kindlin-2 overexpressing cells. BrdU^+^ cells were counted 5 days after transfection and showed a significantly higher percentage of BrdU^+^ cells in Kindlin-2 overexpressing cells compared to control Flag (c and d). DAPI was used as a counterstain. ^*∗*^Significance difference between the indicated groups *p* ≤ 0.05.

**Figure 3:**
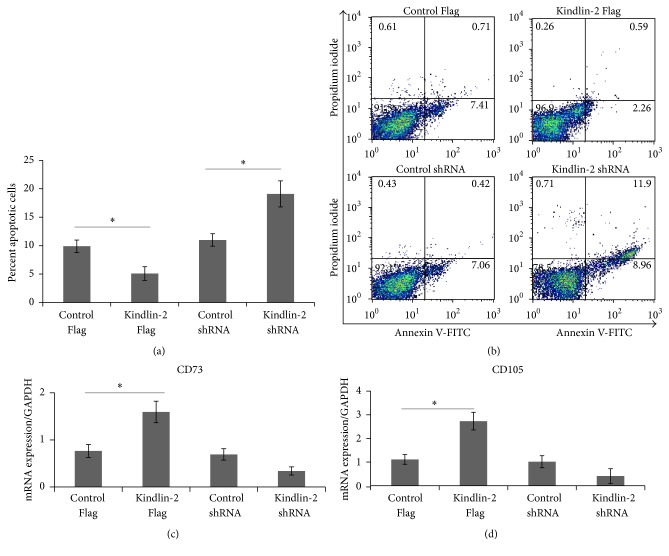
Kindlin-2 regulates apoptosis and stem cell markers in iPSC-MSCs. The iPSC-MSCs were stained using annexin V and propidium iodide for analyzing cell apoptosis after transfection with Kindlin-2 constructs and quantified for early and late apoptotic cells. Kindlin-2 knockdown significantly increased apoptotic cells while Kindlin-2 overexpression significantly decreased apoptotic cells compared to control Flag (a). Data were presented as the mean ± SEM from three independent experiments. Dot plots showing single experiments for annexin V/propidium iodide double positive cells for late apoptotic cells (b). (c) and (d) show quantitative RNA expression of the MSC stemness markers CD73 and CD105 in Kindlin-2 overexpressing and knockdown cells and their controls, respectively. Numbers in (b) indicate % cells in quadrant gate. ^*∗*^Significance difference *p* ≤ 0.05.

Previously, we showed that iPSC-MSCs displayed a shorter doubling time than BM-MSCs and reached senescence at later passages than BM-MSCs [10]. To investigate the effects of Kindlin-2 on proliferation and survival in iPS-MSCs, we performed a BrdU incorporation assay (Figure 2(a)) that showed a significant increase after Kindlin-2 Flag transfection compared to the vector control. In contrast, a small but insignificant decrease in BrdU incorporation was observed after Kindlin-2 shRNA transfection. The same pattern was observed in a WST-1 assay (Figure 2(b)). We confirmed these data by counting the BrdU-incorporated iPSC-MSCs after transfection. The number of BrdU^+^ cells was also significantly higher in Kindlin-2 Flag transfected cells, and there was a minor decrease after Kindlin-2 shRNA transfection compared to control vectors (Figures 2(c) and 2(d)). We next looked for apoptosis via annexin V expression after transfection (Figures 3(a) and 3(b)). The percentage of apoptotic cells was significantly decreased to 3–6% in Kindlin-2 Flag transfected cells when compared to the corresponding control group (7–12%). Moreover, Kindlin-2 knockdown significantly increased the apoptotic cell population in Kindlin-2 shRNA transfected cells to 17–21% (Figures 3(a) and 3(b)). In parallel, expression of the MSC undifferentiated cell markers, CD73 and CD105, was increased in Kindlin-2 overexpressing cells and decreased after Kindlin-2 knockdown (Figures 3(c) and 3(d)).

### 3.3. Differentiation Potential after Kindlin-2 Overexpression in iPSC-MSCs

**Figure 4:**
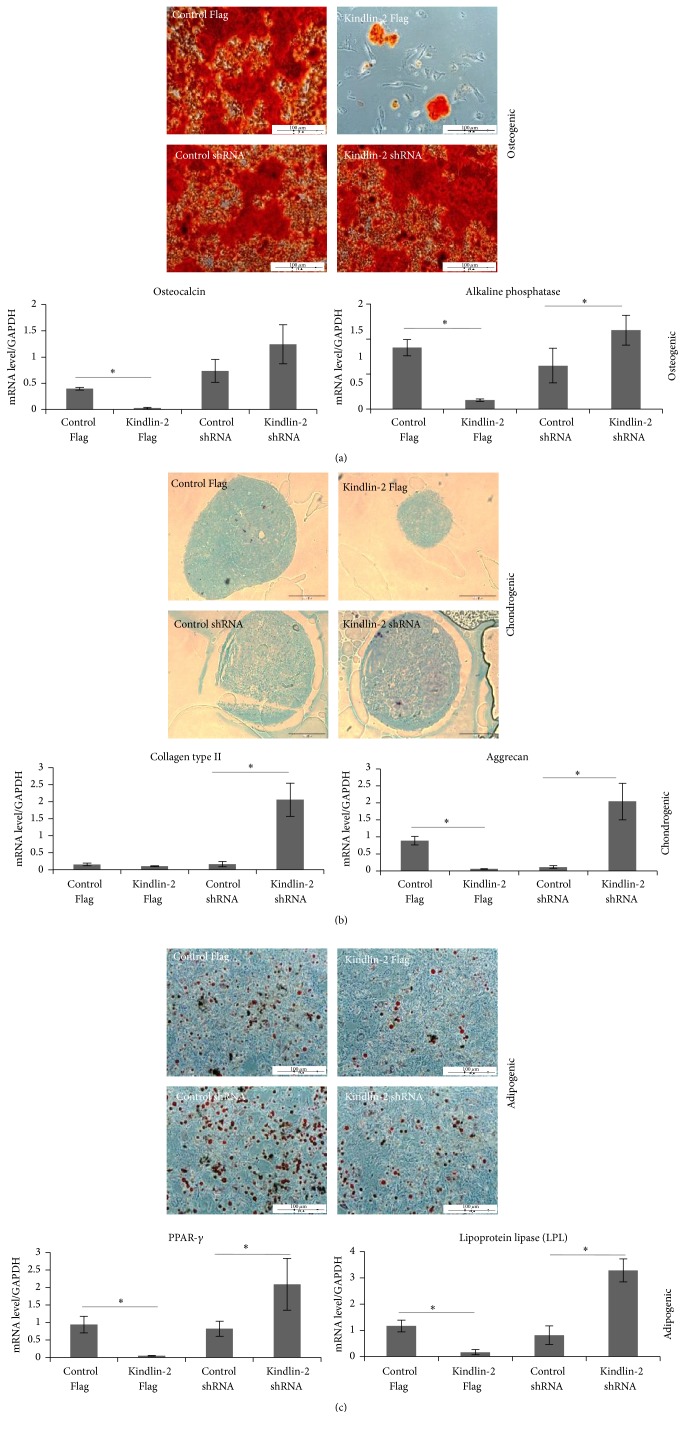
Differentiation capacity of iPSC-MSCs after Kindlin-2 transfection. Osteogenic, chondrogenic, and adipogenic differentiation potential of hiPSC-MSCs after transfection with constructs modulating Kindlin-2. Alizarin Red staining for mineralized deposits (a), Alcian Blue staining for chondrocyte pellets (b), and Oil Red-O staining for lipid formation (c) formed by the three iPSC-MSC cell lines. The mRNA expression level of the relative expression of genes associated with osteogenesis (osteocalcin and alkaline phosphatase), chondrogenesis (collagen type II and aggrecan), and adipogenesis (PPAR-*γ* and LPL). The data represent the mean expression values normalized to the housekeeping gene GAPDH. Magnification is 10x. ^*∗*^Significance difference *p* ≤ 0.05.

To investigate whether the cells may have various differentiation capabilities after transfection, we performed gene expression analysis and morphological analysis on iPSC-MSCs cultured for 21 days under various conditions. During osteogenic differentiation, we detected less calcium accumulation in Kindlin-2 Flag transfected iPSC-MSCs with significantly lower expression of osteocalcin and alkaline phosphatase, whereas expression of alkaline phosphatase was significantly (*p* ≤ 0.05) higher in Kindlin-2 shRNA transfected cells compared to control vector (Figure 4(a)). In chondrogenic differentiation, Kindlin-2 Flag transfected cells produced a small and undifferentiated cartilage pellet; however, the other groups created a larger and fully differentiated cell pellet observed in histological analysis (Figure 4(b)). Furthermore, Kindlin-2 overexpression significantly (*p* ≤ 0.05) decreased aggrecan mRNA levels while cells transfected with Kindlin-2 shRNA had significantly (*p* ≤ 0.05) higher expression of aggrecan and collagen type II (Figure 4(b)). We observed increased numbers of lipid droplets in Kindlin-2 shRNA transfected iPSC-MSCs and a significantly higher expression (*p* ≤ 0.05) of PPAR-*γ* and LPL, while the number of lipid droplets was decreased in iPSC-MSCs transfected with Kindlin-2 Flag vector with significantly lower expression (*p* ≤ 0.05) of PPAR-*γ* and LPL (Figure 4(c)).

### 3.4. Adhesion of iPSC-MSCs under Shear Stress

**Figure 5:**
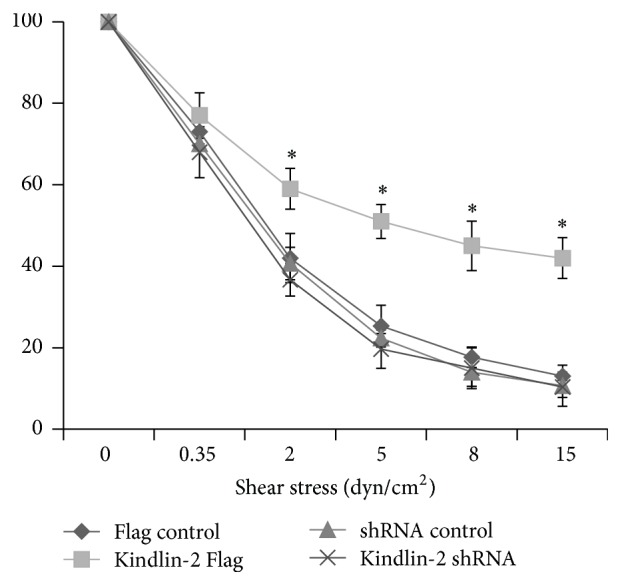
Kindlin-2 boosts iPSC-MSCs adhesion to slides coated with VCAM-1 + SDF1*α*. The iPSC-MSCs transfected with Kindlin-2 Flag and shRNA adhered to the flow chamber slide coated with VCAM-1/SDF-1*α* under shear stress. Kindlin-2 Flag transfected iPSC-MSCs had significantly higher attachment to the slides cocoated with VCAM-1 + SDF-1*α* under shear flow velocities of 0, 2, and 5 dynes/cm^2^ with 10x magnification compared to control Flag. The values shown are mean ± SEM of three independent experiments. ^*∗*^Significance difference against Flag control *p* ≤ 0.05.

To investigate how iPSC-MSCs interact with a relevant homing receptor that activates integrins under shear flow, a parallel plate flow chamber system was used with VCAM-1 + SDF-1*α* coating. We assessed adhesion of iPSC-MSCs and determined the percentage of their capability to remain adherent in different shear stresses from low (0.35 dynes/cm^2^) to high (15 dynes/cm^2^) levels. Determination of numbers of adherent cells at each shear stress indicated that Kindlin-2 Flag transfected cells had a higher affinity to adhere to VCAM-1 + SDF-1*α* coated surface than control vector transfected cells. As shown in Figure 5, after increasing the shear stress to 2 dynes/cm^2^, 59 ± 5% of the Kindlin-2 Flag transfected cells remained attached to the surface compared to 42 ± 6% of the control vector and 36 ± 6% of Kindlin-2 shRNA transfected cells. A more prominent difference was observed after increasing the shear stress to 5 dynes/cm^2^ in which on average 51 ± 4% of Kindlin-2 Flag transfected cells remained attached, whereas control cells declined to 25 ± 5%, and the cells in the Kindlin-2 shRNA group declined to 19 ± 4%. In parallel, 25.5 ± 4.2% of Kindlin-2 Flag cells were also found positive for CXCR4 by flow cytometry, compared to 0.052 ± 0.05% in control vector, 0.02 ± 0.01% in control shRNA, and 0 ± 0% in Kindlin-2 shRNA iPS-MSCs, respectively (means + SD; *n* = 3; not shown in figure). Thus, Kindlin-2 overexpression significantly (*p* ≤ 0.05) increased the adhesion to VCAM-1 + SDF1*α* coated flow chamber slides at shear stresses between 2 and 15 dynes/cm^2^, along with an increased expression of CXCR4.

### 3.5. Migration Potential of iPSC-MSCs after Kindlin-2 Overexpression

**Figure 6:**
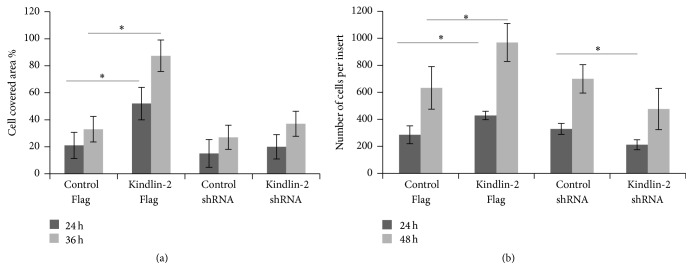
Kindlin-2 affects migration potential of iPSC-MSCs. An in vitro migration assay was performed with an IBIDI culture insert containing two reservoirs separated by a 500 *μ*m thick wall. Kindlin-2 Flag transfected iPS-MSCs had significantly increased migration potential after 24 h and 36 h compared to control groups (a). Moreover, the migration assay with a transwell system showed significantly increased migrating iPSC-MSCs on the lower face of the filters after 48 h of incubation in Kindlin-2 Flag transfected iPSC-MSCs compared to control Flag (b). The covered area between the two reservoirs was analyzed with ImageJ software. ^*∗*^Significance difference *p* ≤ 0.05. *n* = 3.

We next investigated the migration potential of iPSC-MSCs after transfection with the Kindlin-2 constructs using transwell culture inserts. We found a significant difference in the number of transmigrated cells both at 24 h and at 36 h between Kindlin-2 Flag transfected cells (52 ± 13% versus 20 ± 11% after 24 h and 86 ± 11% versus 37 ± 7% after 36 h). However, these differences were not significant between Kindlin-2 shRNA and control groups (Figure 6(a)). We observed that, at 24 h after transfection, 429 ± 31 cells migrated to the lower surface of the culture insert in Kindlin-2 Flag transfected iPSC-MSCs, but only 285 ± 66  cells were counted in the control group (*p* ≤ 0.05). Moreover, the numbers of cells that migrated decreased to 212 ± 37 in Kindlin-2 shRNA transfected cells, which was significantly lower (*p* ≤ 0.05) than the control group. Kindlin-2 Flag transfected cells also migrated significantly better (*p* ≤ 0.05) than cells in the control group after 48 h of incubation (969 ± 140 cells for Kindlin-2 Flag versus 633 ± 157 cells for control). Although we observed a reduced number of migrating cells in the Kindlin-2 shRNA group compared to the control group, this difference was not statistically significant (Figure 6(b)). In conclusion, Kindlin-2 levels resulted in modulated migration of iPS-MSCs.

### 3.6. Kindlin-2 and Anti-Inflammatory Effects of iPSC-MSCs

**Figure 7:**
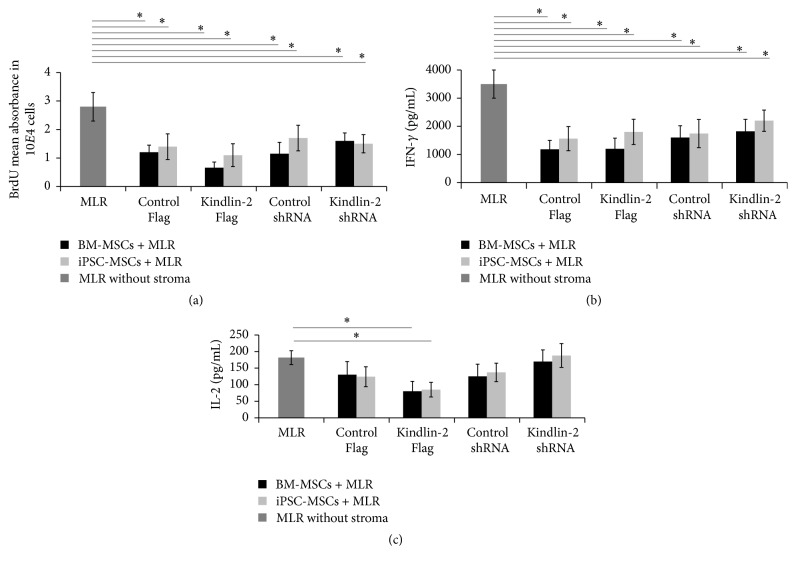
iPSC-MSCs suppress inflammatory reaction after transfection with Kindlin-2. To investigate the immunomodulatory properties of iPSC-MSCs after Kindlin-2 overexpression/knockdown, mixed lymphocyte reaction (MLR) was used to mimic an inflammatory reaction by mixing CD4^+^ lymphocytes with healthy donor peripheral blood mononuclear cells (PMNCs) on iPSC-MSCs or BM-MSCs feeder layers. (a) CD4^+^ lymphocyte proliferation in an MLR assay using BrdU incorporation. The iPSC-MSCs/BM-MSCs could significantly (*p* ≤ 0.05) decrease lymphocyte proliferation in all four groups compared to MLR without a feeder layer. No significant differences were found between the different MSC groups (not indicated). (b and c) Production of cytokines IFN-*γ* and IL-2 in the supernatant of MLR assays from the iPSC-MSC/BM-MSCs coculture experiments. A significant decrease of IFN-*γ* levels was observed between controls without MSCs and iPSC-MSC or BM-MSCs coculture experiments in all four groups, but no significant differences were noted between MSC groups (b). Compared to MLC without MSCs, IL-2 significantly decreased only in the Kindlin-2 overexpression group and not in other groups compared to MLR without a feeder (c). ^*∗*^Significance difference *p* ≤ 0.05.

Previously, we have shown that iPSC-MSCs exhibit potent immunomodulatory function in a mixed lymphocyte culture (MLR) assay by decreasing CD4^+^ T-lymphocyte proliferation and decreasing IFN-*γ* secretion. In this study, we used Kindlin-2 Flag/shRNA transfected iPSC-MSCs and BM-MSCs to determine whether Kindlin-2 expression levels may differentially affect the immunomodulatory properties of these cells in an MLR assay. Overall, our results indicate that there was no significant difference in Kindlin-2 Flag/shRNA expressing iPS-MSCs compared to corresponding controls. However, all groups of iPSC-MSCs as well as BM-MSCs could significantly reduce numbers of CD4^+^ T-cells or release of IFN-*γ* compared to MLR without the feeder layer (Figures 7(a) and 7(b)). However, there were no significant differences in released IFN-*γ* in all MLR assays between iPSC-MSCs or BM-MSCs used as feeder cells (Figure 7(b)). Only Kindlin-2 expressing iPSC-MSCs or BM-MSCs were able to significantly decrease in IL-2 secretion, while the other 3 groups did not show statistically significant differences with the control group (Figure 7(c)).

## 4. Discussion

### 4.1. Kindlin-2 Expression during Derivation of iPSC-MSCs

This study shows that Kindlin-2 is an integrin-associated protein that can alter the phenotype of iPSC-MSCs in terms of proliferation as well as adhesive and migratory properties towards a more primitive phenotype and in a way that is desirable for MSCs to be used as cellular therapeutics [14, 15]. It has been previously reported that Kindlin-2 can regulate cell-cell and cell-ECM adhesion as well as cell migration via integrin and integrin-linked kinase (ILK) activation [16–18]. Kindlin-2 has been shown to play a crucial role in modulation of integrin signaling and activation, which assists the cell in sensing and interacting with the surrounding environment [19]. Kindlin-2 not only modulates inside-out signaling by interacting with integrin *β* chains [20], but also contributes to outside-in signaling by binding to integrin-linked kinase [21]. More recently, it has been shown that Kindlin-2 can regulate integrin *β*1 protein expression in adult cardiomyocytes [22]. Kindlin-2 is also involved in regulating cancer cell invasions with varying functions in different cancer types [23].

Generally, affinity regulation but not regulation of integrin expression levels influences integrin activation. Although a variety of targets of Kindlin-2 such as the MIR-200 family and migfilin have been already described [24, 25] as mediators for enhanced adhesion, migration, and invasion, these effects remained controversial because Kindlin-2 overexpression/knockdown is not always the same in different cell types. For instance, Kindlin-2 overexpression promotes prostate cancer stem cell proliferation [26], whereas it reduces both cell division in colorectal cancer cells [6] and mesenchymal cancer invasion [27]. The use of a Kindlin-2 mutant with a defect in binding to integrin could further clarify the involvement of integrin binding in the observed functions of Kindlin-2 in our study.

We hypothesized that if Kindlin-2 increased proliferation, adhesion, and migration in iPS-MSCs, studying this protein might provide highly proliferative MSCs that can retain or outperform the therapeutically desired functional characteristics of normal MSCs and might address their deficiencies [28, 29]. Our data indicated that Kindlin-2 was expressed in BM-MSCs at significantly higher levels than the iPSCs that we used to differentiate iPSC-MSCs (Figure 1(a)). During embryonic development, Kindlin-2, which is weakly expressed in embryonic stem cells, begins to accumulate at increased levels in mesodermal-derived tissues [3, 30, 31]. Furthermore, the expression of Kindlin-2 increases as epithelial-shape embryonic stem cells proceed through epithelial-mesenchymal transition (EMT) towards mesodermal-differentiated cells [32]. As shown in Figure 1(a), newly differentiated iPSC-MSCs expressed relatively low amounts of Kindlin-2 mRNA and protein. However, this expression increased in later passages.

### 4.2. Kindlin-2 Targets the CXCR-4/SDF-1*α* Axis

We also showed that alteration of Kindlin-2 expression in iPS-MSCs (Figure 1(b)) positively influenced expression of the newly identified Kindlin-2 target, CXCR-4. This indicates that Kindlin-2 may increase cell adhesion and migration through increasing expression of CXCR4 or its availability at the cell surface [33]. Engineered MSCs with high expression of CXCR-4 were shown to have an enhanced migration capacity and also homing ability in irradiated mice, which is related to SDF-1*α* levels inside the bone marrow [34]. Moreover, the CXCR-4/SDF-1*α* axis had a major influence on MSCs recruitment to tissues, chemotaxis, and homing [35].

Increased levels of CXCR-4 in iPSC-MSCs due to Kindlin-2 overexpression may be the key factor in the boosted adhesive capability of the cells to VCAM-1/SDF-1*α* coated flow chamber slides under high levels of shear stress and also better migratory potential. We performed an adhesion assay with VCAM-1 and fibronectin, which were not affected by Kindlin-2 overexpression/knockdown (Supplementary Figure 1 in Supplementary Material available online at https://doi.org/10.1155/2017/7316354). Our findings support previous studies that indicated that enhanced CXCR-4 expression could lead to improved migration potential and MSCs adhesion in the affected sites [36–38].

### 4.3. Kindlin-2 Overexpression Upregulates Proliferation in iPSC-MSCs

Yet, there is still controversy about the influence of Kindlin-2 on cell proliferation. Previously, Kindlin-2 was known as a mitogen-activating protein that led to enhanced proliferation [for a review, see [39]]. However, recently, it has been demonstrated that Kindlin-2 overexpression does not always amplify proliferation but decreases cell division [6, 27]. To this end, we showed here that Kindlin-2 overexpression led to higher proliferation in iPSC-MSCs, whereas its knockdown increased apoptosis. Regarding MSCs infusion therapies, MSCs with enhanced proliferation, migration, and homing ability (adhesion) are favorable and have long-term beneficial effects on the healing of injured areas [40]. So far, our Kindlin-2 Flag transfected iPSC-MSCs are approaching the characteristics of “superfunctional” MSCs as a suitable substitute for normal MSCs in cell infusion therapies. However, it is still necessary to transplant them and investigate their tumorigenic potential.

### 4.4. Kindlin-2 Overexpression Downregulates Multilineage Differentiation of iPSC-MSCs

We have described the intact differentiation potential and the immune suppressive abilities of iPSC-MSCs in a previous study [10]. Here, we found that Kindlin-2 knockdown rendered iPSC-MSCs more prone to differentiation into three mesodermal lineages, whereas Kindlin-2 overexpression dampened these processes. Recently, Wu et al. showed that Kindlin-2 knockdown in differentiated cells, such as chondrocytes, reduced their density by inhibiting TGF-*β*1-induced Smad-2 phosphorylation, which led to lower cell doubling rates and increased apoptosis. Those results are in line with our findings [41]. However, Kindlin-2 knockdown mice in this previous study lacked primary ossification centers and differentiated chondrocytes due to increased apoptosis in primary mesenchymal progenitors in vivo [41]. Our data indicates that, after Kindlin-2 Flag transfection, the differentiation affinity of iPSC-MSCs is significantly diminished using both morphology and differentiation markers, whereas Kindlin-2 knockdown increased the differentiation potential of iPSC-MSCs in vitro.

### 4.5. Kindlin-2 Overexpression Did Not Change Immunomodulatory Effects of iPSC-MSCs

The immune suppressive capacity of iPSC-MSCs and BM-MSCs remained intact after Kindlin-2 overexpression/knockdown in our study. The capability to induce immune tolerance of pluripotent stem cell-derived MSCs and BM-MSCs is one of the fundamental criteria that makes them a promising source for cell transplantation therapies in graft versus host disease (GVHD) [42, 43]. Recently, Cheng et al. have shown reduced proliferation of CD4^+^ and CD8^+^ T-cell populations along with a reduction in the proinflammatory cytokines IFN-*γ* and IL-2 and an increased number of regulatory T-cells after transplantation of pancreatic islets with iPSC-MSCs [44]. We previously showed that iPSC-MSCs could suppress immune reactions the same as BM-MSCs [10]. In the current study, after Kindlin-2 overexpression and knockdown, iPSC-MSCs could still significantly reduce the number of CD4^+^ T-cells (Figure 7(a)) and proinflammatory cytokines (IL-2 and IFN-*γ*) secretion (Figures 7(b) and 7(c)). This indicates that Kindlin-2 overexpression and knockdown did not eliminate the beneficial role of iPSC-MSCs and BM-MSCs in suppressing the immune reaction.

## 5. Conclusion

Based on our hypothesis, we found that Kindlin-2 overexpression increased the proliferative potential of iPSC-MSCs with less apoptosis and enhanced their migration potential and adhesion to VCAM-1/SDF-1*α* under shear stress by increasing the expression of CXCR-4. We also showed that Kindlin-2 overexpression decreased the ability of iPSC-MSCs to differentiate into the adipogenic, osteogenic, or chondrogenic lineages, while maintaining stemness markers. Our findings indicated that the functional ability of iPSC-MSCs to reduce the proliferation of CD-4^+^ T-cells and decrease proinflammatory cytokines (IFN-*γ* and IL-2) in an MLR assay is still intact and generally not affected by Kindlin-2 modification. Together, our data suggest that targeting Kindlin-2 in iPSC-MSCs opens a new way towards cell-therapeutic approaches employing functionally enhanced MSCs.

## Supplementary Material

Supplementary figure 1. The iPSC-MSCs transfected with Kindlin-2 Flag and shRNA adhered to the flow chamber slide coated with fibronectin and VCAM-1 under shear stress. There was no significant difference between different groups and their corresponding controls. The values shown are mean ± SEM of three independent experiments.
