# Supplementary figures and images for: Kindlin-2 Modulates the Survival, Differentiation, and Migration of Induced Pluripotent Cell-Derived Mesenchymal Stromal Cells
Source: Stem Cells Int. 2017 Jan 9;2017:7316354. doi: 10.1155/2017/7316354 (PMC5253493; doi:10.1155/2017/7316354)

Supplementary figure 1

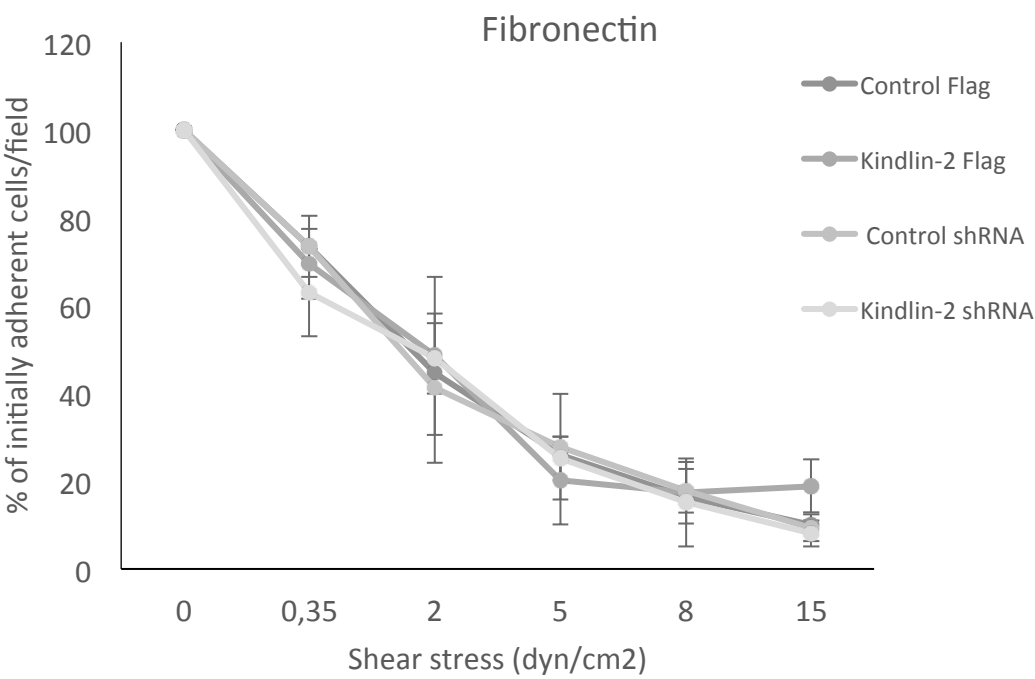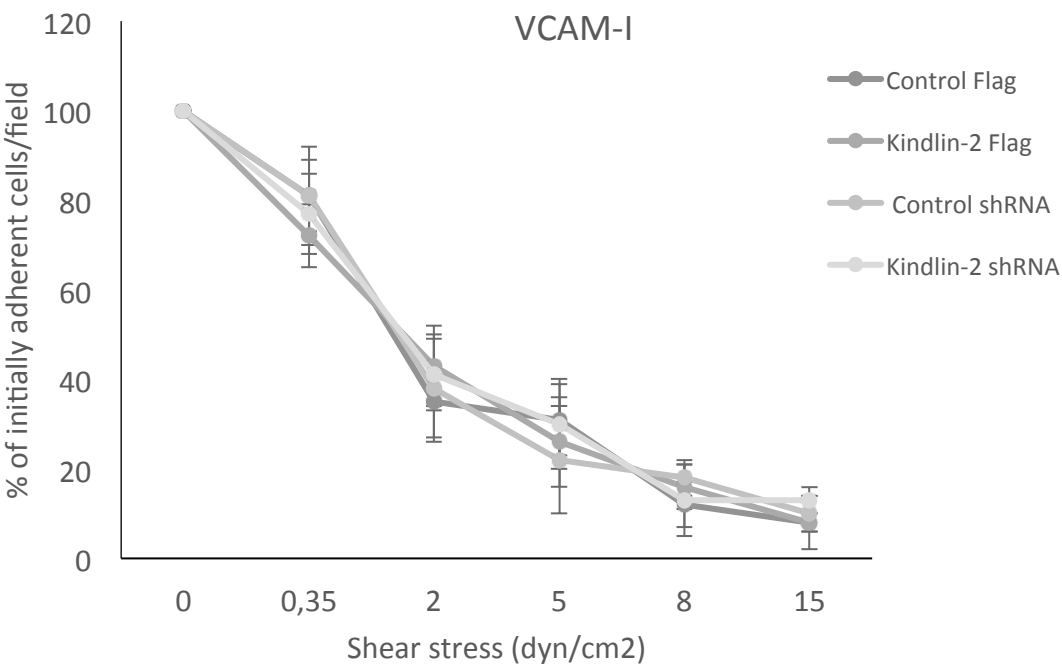

Supplement: Supplementary file 1 — Supplementary figure 1. The iPSC-MSCs transfected with Kindlin-2 Flag and shRNA adhered to the flow chamber slide coated with fibronectin and VCAM-1 under shear stress. There was no significant difference between different groups and their corresponding controls. The values shown are mean ± SEM of three independent experiments. [file 7316354.f1.pdf]
